# An American Text-Book of Physiology

**Published:** 1898-05

**Authors:** 


AN AMERICAN TEXT-BOOK OF PHYSIOLOGY. By Henry P. Bowditch, M. D., John G. Curtis, M. D., Henry H. Donaldson, Ph. D., W. H. Howell, Ph. D., M. D., Frederic S. Lee, Ph. D., Warren P. Lombard, M. D., Graham P. Lusk, Ph. D., W. T. Porter, M. D., Edward I. Reichert, M. D., and Henry Sewall, Ph. D., M. D. Edited by William H. Howell, Ph. M. D., Professor of Physiology in Johns-Hopkins University, Baltimore, Md. Fully illustrated. Published by W. B. Saunders, 925 Walnut street, Philadelphia. 1896. Cloth, $6. By subscription only.

This work, in the manner of its preparation, is to a certain extent an experiment, as all former text-books on physiology have been prepared by individual authors.

No other branch of our science, however, has made more rapid strides in progress than physiology, and as a consequence the literature of the subject has been very great, in recent years. It is also true that the established facts constitute a great mass of information, each division of which needs the discriminating attention of a specialist. It would therefore seem to be a foregone conclusion, that the collaboration method would prove superior to the former method of individual authority. In our judgment this reasonable assumption has been demonstrated to be true in the work before us. As has been previously stated, the progress in physiology has been rapid and important, and therapeutics based on physiology as it was taught ten or fifteen years ago, is faulty, to say the least of it, in many important instances. It therefore behooves the up-to-date physician to have in his library for constant reference the latest authoritative work on physiology. We do not hesitate to say that this is one of the best, if not the very best, yet published. It comprises 1003 pages, including a full index, is handsomely illustrated and clearly printed. It is divided into fourteen chapters, the first six of which are written by the editor, William H. Howell, under the following titles: Introduction, General Physiology of Muscle and Nerve, Secretion, Chemistry of Digestion and Secretion Movements of the Alimentary Canal, Bladder and Ureter, and Blood and Lymph. The chapter on the Circulation is written by W. T. Porter; Respiration and Animal Heat, by Edward I. Reichert; the Central Nervous System, by Henry H. Donaldson; the Special Senses, by Henry P. Bowditch; Physiology of Special Muscular Mechanisms, by Henry Sewall; Reproduction, by Frederick S. Lee, and the Chemistry of the Animal Body, by Graham Lusk.

Each and every article is ably written, and the bibliography of the subject is freely referred to, an unusual feature in a text-book. Howell's Physiology will be an acquisition of value in any physician's or student's library. I. J. J.

